# High Power Generation with Reducing Agents Using Compost Soil as a Novel Electrocatalyst for Ammonium Fuel Cells

**DOI:** 10.3390/nano12081281

**Published:** 2022-04-09

**Authors:** Verjesh Kumar Magotra, Seung Joo Lee, Tae Won Kang, Akbar I. Inamdar, Deuk Young Kim, Hyunsik Im, Hee Chang Jeon

**Affiliations:** 1 Nano Information Technology Academy, Dongguk University, Jung-Gu, Seoul 100715, Korea; birju.srm@gmail.com (V.K.M.); leesj@dongguk.edu (S.J.L.); twkang@dongguk.edu (T.W.K.); 2 Division of Physics and Semiconductor Science, Dongguk University, Jung-Gu, Seoul 100715, Korea; akbarphysics2002@gmail.com (A.I.I.); dykim@dongguk.edu (D.Y.K.); hyunsik7@dongguk.edu (H.I.)

**Keywords:** fuel cell, compost soil, electrocatalysis, ferricyanide, ammonium fuel

## Abstract

Ammonium toxicity is a significant source of pollution from industrial civilization that is disrupting the balance of natural systems, adversely affecting soil and water quality, and causing several environmental problems that affect aquatic and human life, including the strong promotion of eutrophication and increased dissolved oxygen consumption. Thus, a cheap catalyst is required for power generation and detoxification. Herein, compost soil is employed as a novel electrocatalyst for ammonium degradation and high-power generation. Moreover, its effect on catalytic activity and material performances is systematically optimized and compared by treating it with various reducing agents, including potassium ferricyanide, ferrocyanide, and manganese dioxide. Ammonium fuel was supplied to the compost soil ammonium fuel cell (CS-AFC) at concentrations of 0.1, 0.2, and 0.3 g/mL. The overall results show that ferricyanide affords a maximum power density of 1785.20 mW/m^2^ at 0.2 g/mL fuel concentration. This study focuses on high-power generation for CS-AFC. CS-AFCs are sustainable for many hours without any catalyst deactivation; however, they need to be refueled at regular intervals (every 12 h). Moreover, CS-AFCs afford the best performance when ferricyanide is used as the electron acceptor at the cathode. This study proposes a cheap electrocatalyst and possible solutions to the more serious energy generation problems. This study will help in recycling ammonium-rich wastewaters as free fuel for running CS-AFC devices to yield high-power generation with reducing agents for ammonium fuel cell power applications.

## 1. Introduction

Nowadays, energy consumption and population growth have increased the demand for cheap, clean, green, and affordable energy. Moreover, eutrophication and chemicals such as ammonia and ammonium are serious environmental pollutants [1], without neglecting other sources of industrial waste nitrogen, such as coke-plants, tanneries, textiles, landfill leachate, and fertilizer wastewater. Thus, the removal of nitrogen from wastewater is becoming an increasingly urgent concern for saving human and aquatic life. Various methods are being developed to eliminate ammonium ion from polluted water [2]. The simultaneous energy production and degradation of contaminants in wastewater affords an economic and environmental alternative for producing clean and green energy from these wastes [3,4,5]. Recently, hybrid microbial fuel cell (MFC) devices have been used to clean the environment and generate efficient and sustainable energy [4,5]. They have various applications such as hydrogen production; cleaning ammonium-, urea-, and urine-rich wastewater; biosensors; stripping; ion exchange; adsorption; membrane processes; and microbial electrosynthesis. Many electrocatalysts are currently utilized in the market for cleaning wastewater [6,7]. In conventional cleaning methods for removing ammonia and ammonium from wastewater landfills, leachate saline wastewater necessitates the use of precious electrocatalysts such platinum, boron-doped gold, diamond, palladium, and iridium oxide [8,9]. However, these catalysts have numerous issues: they are costly, have deactivation problems, and not stable for ammonium oxidation applications for long-term use at a large scale [10,11]. Therefore, chemical methods by precipitation or oxidation methods and biological process are employed in the nitrogen cycle and wastewater treatment, where thousand types of microorganisms comprising ammonium-oxidizing bacteria (AOB) and biological nitrogen removal (BNR) take part in the process [12]. Although compost soil can beneficially degrade ammonia/ammonium in the soil via the oxidation process, decomposition through natural processes is time consuming [4,13,14]. Compost soil is the best electrocatalyst reported in previous studies that is cheap and sustainable [2,4,15,16,17]. Moreover, as is well known, the power performance of MFCs depends to a large degree on the following factors: the substrate, exoelectrogenic microorganisms, circuit resistance, electrode material, fuel, and the electron acceptor [18,19,20,21]. Furthermore, all reducing agents possess different physical and chemical properties, which directly affect the power production efficiency of MFCs. The applicability of new electron acceptors in MFCs, which significantly impacts electricity generation, has recently gained attention [21,22]. The application of alternative electron acceptors may enhance and increase power generation, reducing the operating and maintenance costs of MFCs and expanding the scope of current and large-scale future renewable energy applications. Current research findings suggest that compost-based MFCs can control environmental pollutants through soil processes and consequently enhance the power performances of compost soil ammonium fuel cells (CS-AFCs) [23,24]. For example, ammonia, ammonium, and nitrate are well-known pollutants present in the environment.

Compost soil has its own importance and scope as a nanoparticle material present in the natural environment, being made up of superdispersed particles in the air and biological objects such water and soil colloids, as well as materials constructed using nanomaterial. Soil colloids have been studied for many decades [25]. Therefore, their surface properties (e.g., physical, chemical, electrical, and reactivity) have become more important in biogeochemical processes and are even dominant in relation to their mass properties. Moreover, as more and more applications of soil nanoparticles have been found in materials, increasing attention is being paid to the properties of those nanoparticles in the power generation sector [26,27,28].

Different types of soils, such as normal soil carbon-rich compost soil, garden soil, and nitrogen-rich compost soil, are abundant on the earth. Due to aerobic/anaerobic bacterial behavior in soil, soil has a complex nature [24,29,30]. Ammonium chloride was previously known only as an ionic conductor, source of nitrogen, and used as fuel, but it generates very minute electricity via aerobic/anaerobic interactions with specific bacteria such as *Nitrosomonas Europaea* [2,4]. However, to our knowledge, no study has employed compost soil as an electrocatalyst with different reducing agents at the cathode, namely (1) ferrycyanide, (2) ferrocyanide, and (3) manganese dioxide. Herein, compost soil is used as a new and cheap electrocatalyst, achieving the goal for ammonium detoxification and high-power generation for CS-AFCs [4,31,32].

Soil, membranes, pollutants, compost, and reducing agents were all used in this current piece of research. All of these can be directly related to energy concerns. In terms of nano energy and nanomaterials, this field is interesting. Due to the role of soil processes in a device for a particular study in an alkaline medium [33,34], soil properties are understood to be well suited to the catalytic reaction for active molecules applied in CS-AFCs. A comprehensive fundamental understanding of alkaline-based reactions in CS-AFCs was emphasized and this falls inside aims and scope of nanomaterials.

## 2. Experimental

### 2.1. Sample Preparation and Fabrication of Fuel Cell

For the testing and fabrication of the fuel cell device and many materials used for the main paperwork, copper (Cu/Cu) electrodes were selected, and later, for comparison, lifelong stability period graphite (G/G) electrodes were used [35]. The following composts, supplied by Bukyong Global Co., Gyeonggi-do, Seoul Korea, were used: (1) carbon-rich compost (2), cow-dung-based compost. After basic testing without membrane, studies on membrane reduction led to the carbon-rich compost soil being selected for further reduction studies for CS-AFCs [35]. Reducing candidates such as ferricyanide, ferrocyanide, and manganese dioxide were supplied by Duksan reagents company, Seoul, Korea [36]. First, cells were fabricated using the coin cell assembly type CR2032, which is generally used for Li-ion batteries [37]. An MTI Korea battery, R&D materials, and an MPG-2 16-channel battery cycler (Biologic Scientific Instrument, 4 Rue Vaucanson, 38170 France) were used to conduct cyclic voltammetry studies on the coin cells [38]. A porous poly (vinylidene fluoride) membrane (PVDF) was purchased from Gelman Sciences 66477, Pall Co., Ltd., Beijing, China [39]. The coin cells were assembled in the open atmosphere using a 15-mm copper foil at the anode and cathode loaded with 3 g compost soil [40]. A dilute ammonium synthetic pollutant made from ammonium chloride chemical salt (Duksan reagents, Seoul Korea) was employed. Additionally, different concentrations of ammonium salt were mixed with distilled water (DW) to yield different fuel concentrations of 0.1, 0.2, and 0.3 g/mL of ammonium chloride for basic studies [41]. After optimizing the coin cell studies, a multifunctional big fuel cell CS-AFC device was designed and fabricated for actual application. The CS-AFC device had the following parameters: a surface area of 15 cm^2^ with 50 g of compost soil. At the initial level, 50 mL of fuel was well mixed, and the device was left for some time for activation [42]. For the reference sample, a carbon-rich compost coded with D.W mixed with soil (RCS-AFC) was used once, and compost soil with 0.2 g/mL NH_4_Cl fuel concentration was coded as CS-AFC. Bacterial and autoclaved studies were performed, and the results were analyzed to observe the effect on CS-AFC.

### 2.2. Electrochemical and Electrical Characterization

To study the electrochemical properties of composts, the soils were investigated using a cyclic voltammeter (C–V) in the bipolar mode. Additionally, temperature-dependence studies, electrochemical impedance spectroscopy (EIS), chronoamperometry, cyclic stability studies, and polarization studies were performed. Impedance studies were conducted using the Nyquist plot obtained from the real and imaginary curves obtained from the EIS measurements of the concentration-dependent samples, with these falling in the range of 0–10,000 Hz for an applied alternating signal of 10 V. Chronoamperometry was performed for the 0.2 g/mL NH_4_Cl fuel concentration with a power output of 0.3 V to check for sample durability, where the role of the bacteria in the carbon-rich compost soil CS-AFC sample was compared to the autoclaved sterilization sample of the CS-AFC treated at 121 °C (to kill the bacteria); then, I–V studies were performed to verify the role of the bacteria with the electrocatalyst. Furthermore, the temperature-dependent studies were performed at three different temperatures, 25, 40, and 55 °C, using a Joe-Tech Oven (TC-ME-06) Seoul Korea. As mentioned earlier in the I–V measurement studies, the cells must be refueled to obtain a constant power output and a continuous fuel supply is required for regular performance. The refueling time was calculated considering the ratio of the voltage across the load in the circuit to the maximum output voltage of the cell under the no-load conditions. Owing to the small size of the CS-AFC coin cell, the cell surface area of 3.14 cm^2^ was increased to 15 cm^2^ to make it suitable for future commercial electrical applications [4,43].

## 3. Results and Discussion

### 3.1. Electrochemical Studies

Electrochemical studies of CS-AFCs were performed using a coin cell assembly type CR2032 system. Herein, ammonium chloride was employed as the fuel in various concentrations, 0.1, 0.2, and 0.3 g/mL; to prepare the samples, it was mixed with carbon-rich soil, and then, the samples were analyzed without membrane, with membrane, and with different reducing agents, such as potassium ferricyanide, ferrocyanide, and manganese dioxide, to enhance the device power. Cu foil was used as the current collector electrode at the anode and the same Cu was used for the electron acceptor at the cathode separated with the compost soil accompanied by standard porous bipolar membrane PVDF to avoid any recombination. This ensures its positive effects on the power performance of the CS-AFC with these additional reductions at the cathode. The texture of the compost soil used herein was investigated via SEM (JSM-6701F, JEOL, Japan). (scanning electron microscope measurements at different magnifications (shown in Figure S2a,b from the Supplementary Materials)). Figure S3a,b displays a schematic diagram for the I–V studies performed for the coin cell type as well as for the giant size design for continuous power generation from the CS-AFCs.

**Figure 1:**
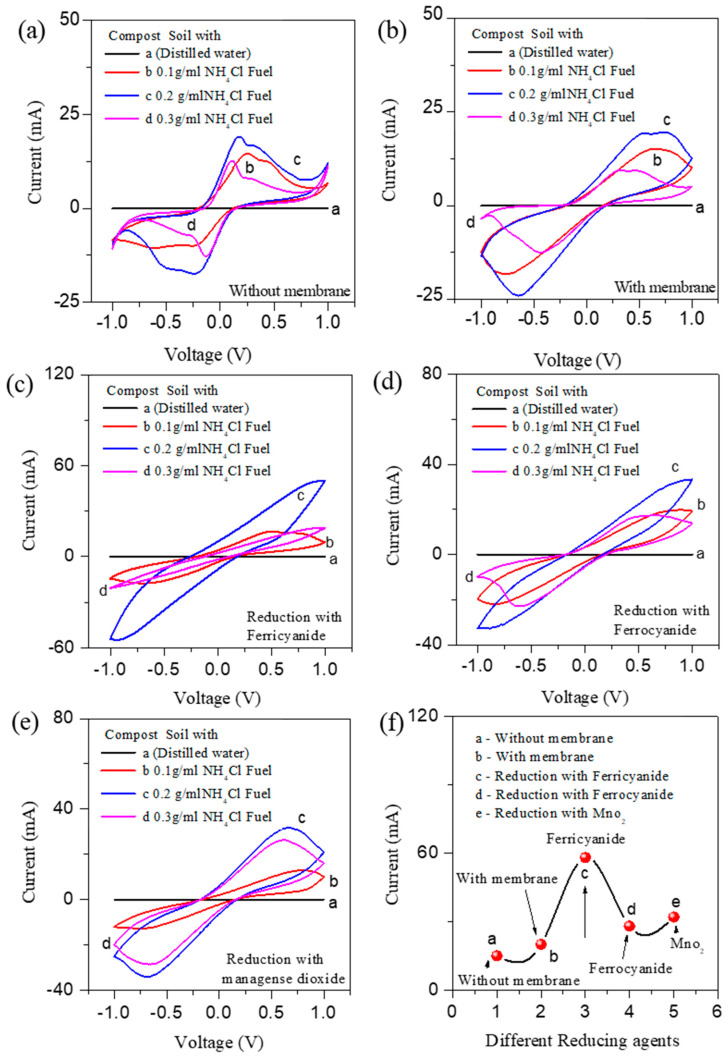
Bipolar C-V studies showing the comparison of varying fuel concentrations in CS-AFCs using reductions: (**a**) without membrane, (**b**) with membrane, (**c**) ferricyanide, (**d**) ferrocyanide, and (**e**) manganese dioxide; (**f**) comparison of the redox potential performance with different reducing agents.

Figure 1a–e shows the C–V bipolar scans measured at a scan rate of 50 mV/s for various CS-AFCs. The C–Vs were recorded in the potential window of ±1.0 V. Herein, different parameters were tuned, i.e., CS-AFCs with and without membrane and CS-AFCs with different reducing agents, such as potassium ferricyanide, ferrocyanide, and manganese dioxide, to realize and optimize the electrochemical performance. The CS-AFC fabricated using DW, as a reference sample, was compared with those fabricated with 0.1, 0.2, and 0.3 g/mL NH_4_Cl fuel concentration. The surface area of the C–Vs for the different NH_4_Cl fuel concentrations indicates that the fuel concentration directly affects the power generation. As is well known, the peak current and onset potential are commonly used to evaluate a catalyst’s electrocatalytic activity towards any electro-oxidation or electro-reduction. The results show that mild oxidation peaks are observed in the DW fuel-based samples due to the natural ammonium component present in the compost. The C–Vs exhibiting higher redox have higher electrocatalytic activity for ammonium fuel oxidation when treated with ammonium chloride fuels at different concentrations [44].

The CS-AFCs comprising 0.2 g/mL NH_4_Cl fuel concentration exhibited the highest catalytic activity among all the studied CS-AFCs. CS-AFCs with and without membrane afford different C–V curve shapes with different oxidation potentials. The oxidation peak observed at 0.5 V generated in the CS-AFC without membrane (Figure 1a) is slightly shifted towards 0.7 V in the CS-AFC with membrane (Figure 1b). The indication of the redox value and current shifting is enhanced because a small amount of nitrate is always present in most soils/composts due to ammonification/nitrification. 

**Figure 2:**
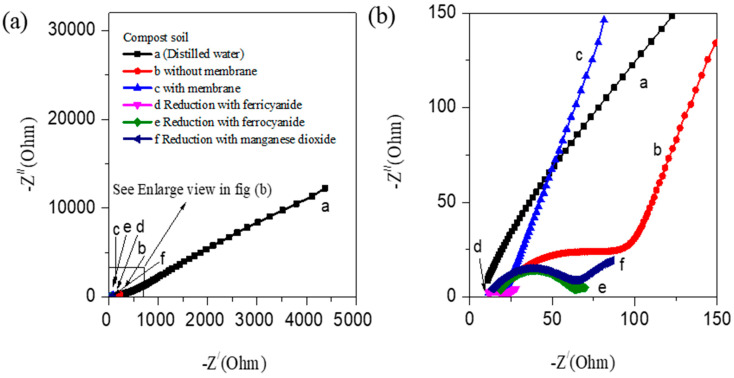
(**a**) EIS study showing the effect of different reducing agents using 0.2 g/mL fuel on different compost samples. (**b**) Enlarged view of the EIS curves.

Figure 1c–e show the different C–V shapes of the CS-AFCs fabricated using potassium ferricyanide, ferrocyanide, and manganese dioxide and the oxidation peak shifting from the ammonium basic ±0.5 V toward ±1 V. The major shift of values in the ammonium oxidation and reduction peaks shows the higher redox potential observed in the potassium ferricyanide compost sample (Figure 1c) as an electron acceptor compared to the ferrocyanide (Figure 1d) and manganese dioxide (Figure 1e) compost samples. The current density is another crucial factor that is estimated from the C–V curves for all the CS-AFCs. The CS-AFCs with potassium ferricyanide (Figure 1f) as a reducing agent afford higher current densities than all other CS-AFC devices. Ammonium chloride acts as an ionic conductor as well as an electron donor for the CS-AFCs [4]. The EIS analysis of all the CS-AFCs supports these results. Figure 2a,b shows the EIS spectra of all the CS-AFCs recorded in the low and high frequency regions (0–10,000 Hz). All the CS-AFCs yielded a semicircle in the low frequency region and a straight line in the high frequency region. Note that the semicircle and straight line are associated with the charge transfer resistance (Rct) and Warburg impedance (W), corresponding to the diffusion coefficient. The CS-AFC with potassium ferricyanide exhibits a smaller Rct (enlarged view in Figure 2b) than all the other CS-AFCs. This could be the possible reason for the high catalytic activity of the CS-AFC with potassium ferricyanide. For an electrode comparison of the catalytic activity, additional C–Vs were recorded at different current collectors, such as Gr/Gr at the anode and cathode. The comparative C–Vs performed at a scan rate of 50 mV/s and the EIS for the cell fabricated without a membrane are shown in Figure S4a,b. The figure shows that the CS-AFC with Cu as the current collector exhibited a higher current density than that with Gr/Gr electrodes, and the oxidation peaks of the Gr/Gr electrodes matched with those of Cu/Cu from the literature. The EIS data support the result that the CS-AFC with Cu as the current collector affords low charge-transfer resistance. Thus, the system is suitable for cleaning environmental toxins while generating power, and the role of the ferricyanide is justified for ammonium fuel cells.

**Figure 3:**
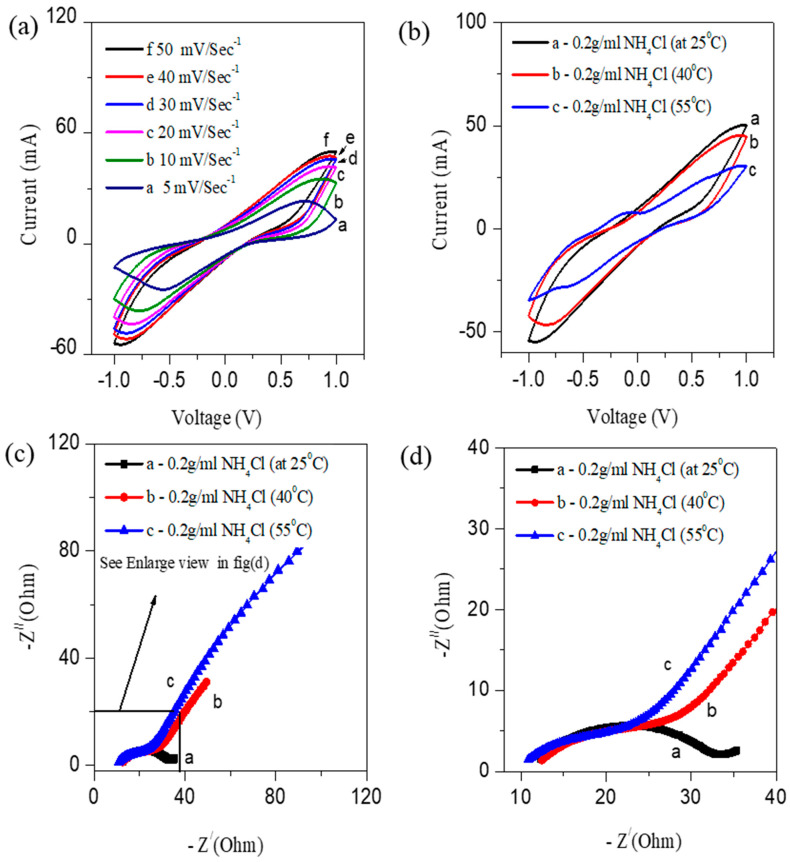
Effect of temperature on the electrochemical properties of CS-AFC with 0.2 g/mL NH_4_Cl fuel concentration with ferricyanide. (**a**) Scan rate variation of the CS-AFC; (**b**) C–Vs recorded at different temperatures. (**c**) EIS data of the temperature dependence of the CS-AFCs. (**d**) Enlarged view of the Nyquist plots.

To ascertain the reversibility of the CS-AFC with potassium ferricyanide, scan rate and temperature-dependent C–V studies were conducted. Figure 3a shows the scan behavior: the current density increases with the voltage, as shown by the C–V curves recorded at different scan rates (5, 10, 20, 30, 40, and 50 mV/s). The bipolar scan rate dependence behavior of the CS-AFC (Figure 3a) sample was studied, focusing on the change in the device behavior from a quasi-reversible to an irreversible state with the increase in the scan rate toward the positive polarity and similarly toward the negative polarity. The linear increase of the current density indicates that the diffusion-controlled power of the fuel cell depends on the scan rate and concentration of the reducing agent, which is similar to typical MFCs. Moreover, temperature dependency studies (25, 40, and 55 °C) were performed to determine the effect of temperature on the device performance. Figure 3b shows the C–Vs measured at different temperatures for the CS-AFC with potassium ferricyanide. The figure shows that the sample’s electrocatalytic activity and redox potential are higher at 25 °C than at 40 and 55 °C. This is supported by the EIS analysis at different temperatures, which showed that higher performance curves have lower resistance and lower performance curves have high impedance, as shown in Figure 3c,d.

**Figure 4:**
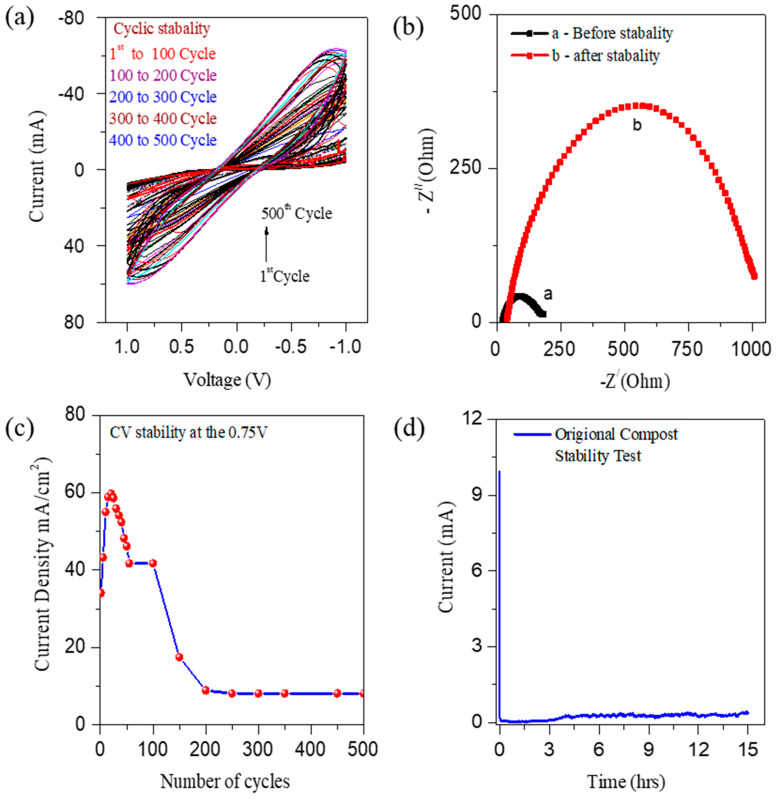
Electrochemical cyclic stability measurements of CS-AFCs with ferricyanide and a single shot of fuel (0.2 g/mL). **(a)** Cyclic stability recorded for 500 cycles. (**b**) EIS study before and after the stability of 500 cycles; (**c**) current density vs. cycle number. (**d**) Chronoamperometry stability for 12 h.

Device stability is also an essential factor for practical use. The best performing CS-AFC, the CS-AFC with potassium ferricyanide as an electron acceptor and 0.2 g/mL NH_4_Cl, was tested for stability measurements using C–V. Figure 4a shows the C–V curves at a scan rate of 50 mV/s and 500 cycles with a single fuel shot. The study was conducted at room temperature. The repeated C–V studies show that compost can be used as a long term electrocatalyst in CS-AFCs. Additionally, the same compost soil can be reused multiple times as an electrocatalyst after refueling. The results show that the cyclic stability of the device is sufficient for multifunctional device application in both the polarities. Figure 4b shows results of the EIS studies before and after the 500 cycle’s stability test. Figure 4c shows the graph of the current density against the cycle number. The figure shows that the current density reaches a maximum of about 60 mA at the 20th cycles, and then it gradually decreases until the 500th cycle. Furthermore, the device stability was investigated using chronoamperometry analysis at 0.3 V (Figure 4d). Chronoamperometry determines the compost sample life’s behavior as an electrocatalyst for the multifunctional fuel cell. First, it reaches a maximum current of 10 mA/m^2^, and then the current slowly decreases due to ammonium oxidation. The device’s total life span is a minimum of 12 h with Cu/Cu electrodes. The use of ferrycyanide as a reducing agent does not affect the stability of the fuel cell. Note that a commercial fuel cell needs to be refueled multiple times for efficient and sustainable device operation for cleaning toxins and electricity generation.

### 3.2. Polarization and Power Studies

**Figure 5:**
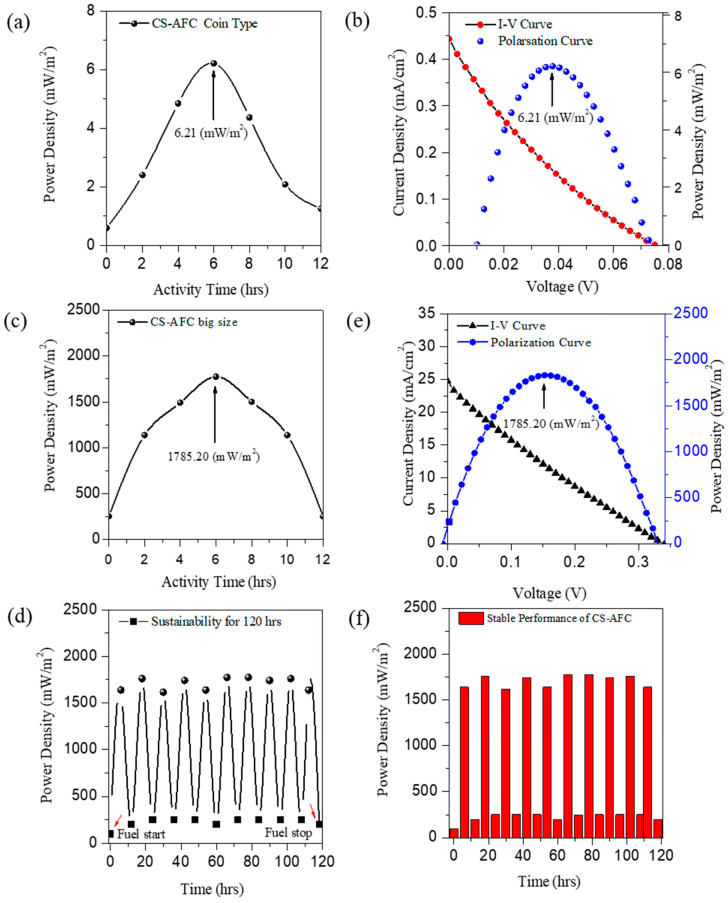
I–V measurement data for the CS-AFC with ferricyanide and 0.2 g/mL fuel concentration. (**a**) Catalytic activity in coin cell with a surface area of 3.14 cm^2^ (1–12 h) cycle. (**b**) Polarization curve of the coin cell. (**c**) Catalytic activity for large-sized CS-AFC. (**d**) Polarization curve of the large-sized CS-AFC. (**e**) Sustainability study of the large-sized CS-AFC. (**f**) Stable performance of the CS-AFC.

To study the electrocatalytic activity of the CS-AFC with ferricyanide and a 0.2 g/mL fuel concentration, I–V measurements were performed between 0 and 12 h. Figure 5a,b shows the power density curves with respect to the operation time. The figure shows that the coin-type CS-AFC standard sample activates around 12 h for catalytic activity, generating the highest peak power (P_max_) at the 6th hour in the single-cycle (Figure 5a). The maximum power density afforded by the coin cell was 6.21 mW/m^2^ (Figure 5b). A similar I–V study was performed for a comparatively bigger fuel cell (Figure 5c,d) for a possible industrial application model; it afforded the highest catalytic activity, polarization, and I–V curves. The maximum power density obtained was 1785.20 mW/m^2^, which is 17 times higher than that reported in previous studies [4,44]. According to the measurements performed using the Keithley SMU unit, the voltage value is low in the mass transport region (i.e., higher current) and the voltage is high in the inactivation region (i.e., low current), which yields low power densities. However, in the ohmic region, the power density is at a maximum value; hence, the power density is symmetrical. Sustainability studies were also performed to optimize the device’s stability by refueling it numerous times at regular intervals, and its long-term sustainability was examined in continuous mode for 120 h, as shown in Figure 5e. Figure 5f shows that the CS-AFC device functions stably while the refueling is continued. It works longer without any deactivation. The experiments were repeated over ten times, and the corresponding standard deviations in the respective results were examined for stable power generation applications. The average and standard deviation of power density were aimed to be 1731.42 ± 67.16 mW/m^2^. The corresponding deviations indicate that our results are repeatable and sustainable for continuous power generation applications. If the CS-AFCs are refueled at regular intervals, they will consistently function. Moreover, to support this study, in Figure S5, the power density table of different fuel cells from the literature is compared to that of our CS-AFCs. The figure confirms the device’s size scalability without any negligible deactivation of the carbon-rich composts acting as a direct electrocatalyst for the CS-AFCs. Additionally, the multifunctional fuel cell has many other advantages: its sustainability, few leakage issues, low maintenance, its efficiency and economy, its availability, and that it is non-toxic, eco-friendly, and abundant [4,35].

### 3.3. Bacterial and Autoclaved Sterilisation Studies

**Figure 6:**
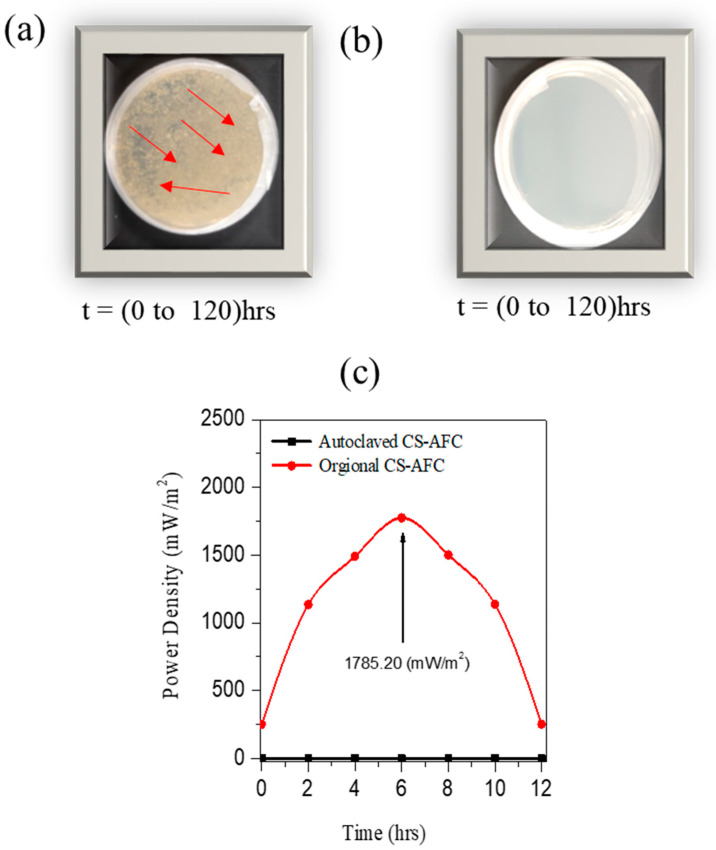
Bacterial study for the CS-AFC samples. (**a**) Growth of the bacterial colonies is present. (**b**) Growth of the colonies is not observed. (**c**) I–V study showing the effect of the bacteria on the power performance.

To verify the authenticity of the result that the electrocatalytic activity observed from the I–V studies and chronoamperometry is due to the ammonium oxidation mechanism based on biological action, we conducted bacterial studies. The bacterial studies demonstrate the role of bacteria and enzymes in the functioning of a standard two-chamber CS-AFC. From the two standard comparison samples, one compost sample was sterilized via autoclave treatment, and its electricity generation was compared with that of a nonsterilized sample. Figure 6a displays the first standard sample, exhibiting the microbial growth of the bacteria. Figure 6b displays the second sample that was autoclaved at 121 °C, exhibiting no microbe growth. Figure 6a,b display a clear picture of the bacterial growth in agar plates between 0 and 24 h: microbe colonies were visible in the plates in Figure 6a and no bacterial growth was observed in the plates of the autoclaved sample shown in Figure 6b. However, ammonium oxidation could have occurred due to microbes that employ biological nitrification/denitrification processes, such as *Nitrosomonas Europaea*. The C–V and EIS analyses of the two samples also prove the effect of autoclave treatment on the CS-AFC samples. Nevertheless, the different types of microbes present in the soil that have complex natures still need to be studied, as the electricity produced by bacteria such as *Nitrosomonas Europaea* has also been scantly reported. Figure 6c shows the Keithley I–V measurements for a standard CS-AFC, affording a maximum power density of 1785.20 mW/m^2^. In comparison, the autoclaved sample had a low power density of 1.2 mW/m^2^ (due to the killing of the bacteria). This further proves the role of microbes in the ammonium oxidation process for power production, exhibiting the effectiveness of compost soil as an electrocatalyst. A comparison of the results obtained from the bacterial and I–V studies shows that bacteria and enzymes play an essential role in electricity generation. Additionally, the actual plates were intermittently photographed, and the bacterial growth was monitored for a long time (up to 120 h), as shown in Figure S6a,b.

The mechanism for the ammonium fuel cell is as follows. The actual mechanistic reaction details of ammonium oxidation remain unexplained to date. However, ammonium may contribute to electricity generation in MFCs in two ways. First, since ammonium is at its lowest oxidation state, it can supply electrons via its oxidation; thus, ammonium may function as an anodic fuel in MFCs. Ammonium oxidation occurs under aerobic and anaerobic conditions, both of which afford negative Gibbs free energy. Consequently, the standard potentials of both the reactions are positive, indicating that they can theoretically generate electric energy in MFCs with ammonium as the electron donor (anode) and nitrite/nitrate or oxygen as the electron acceptor (cathode). Second, ammonium may be utilized by nitrifying and denitrifying bacteria to yield organic compounds that are used by heterotrophs to generate electricity [2,4,42].

The increase or decrease in pH refers to hydroxyl group OH^−^ (pH increases) and H^+^ (pH decrease). Soils can bind ammonium (NH_4_^+^) ion in compost soil and perform ammonification through nitrification and denitrification to reach and release N_2_O–NO, yielding nitrogen (N_2_) as the final product while supplying electrons and protons [2,45]. Thus, ammonium chloride is a source of nitrogen that will be helpful for nitrification and denitrification. Our results demonstrate that power is enhanced when fuel is supplied to CS-AFCs. The concentration studies clearly show the enhancement: 0.2 g/mL fuel concentration affords a stable pH and high performance along with the reduction at the cathode. Due to the addition of nitrogen in the soil, the chemical reaction enhances the pH from 8 to 9.5 (the compost basic range pH is 8 and it reaches 9.5 in the alkaline medium, as reported in the literature). The V_max_ for the high-affinity response reaction (N_2_O → NO → N_2_) showed a relatively small peak, followed by a decline and then a sharp increase. To verify the results, pH experiments were performed, and the pH of all the CS-AFCs. 

Against the days was analyzed for checking the performance sustainability [46]. Hydroxyl ions are also naturally present in compost soil. Furthermore, the nitrogen cycle and ammonia/ammonium-rich wastewater treatment for nitrification and denitrification contain many groups of microorganisms, such as AOB, BNR, *Nitrosomonas Europaea*, and nitrate-reducing bacteria that are helpful for ammonium oxidation [2,4,14,47,48,49,50].

## 4. Conclusions

This study reports, for the first time, that compost can be used as an efficient and sustainable electrocatalyst for ammonium oxidation and energy generation while using different reducing agents as electron collectors. Models were fabricated with different reducing agents to optimize the compost and ammonium fuel concentrations. Among the studied reducing agents, potassium ferricyanide afforded the best performance. Moreover, an ammonium fuel concentration of 0.2 g/mL was optimal. Additionally, the electrocatalytic activity decreased with increasing temperatures; thus, room temperature was determined to be the best for the sustainable cyclic stability of the device and long life of the electrocatalyst. I–V studies confirmed the power generation of a commercial device working as an electrocatalyst; the commercial model afforded a power density of 1785.20 mW/m^2^. Moreover, the power performance improved with the fuel and was directly affected by the device’s size scalability; the role of the reducing agents was also evident. The sustainability studies were performed every 12 h to refuel the device for continuous working. This study presents an electrocatalyst device technology providing eco-friendly, cheap, and sustainable energy generation technology with research scope for soil-based devices.

## Data Availability

The data presented in this study are available on request from the corresponding and first author.

## Supplementary Materials

The following supporting information can be downloaded at: https://www.mdpi.com/article/10.3390/nano12081281/s1. Figure S1. The schematic diagram showing the vision of this paper is shown in Figure S1. This figure highlights the use of the multifunctional device for environmental detoxification and continuous power generation; Figure S2. (a,b) Explain the SEM image to study the surface texture of the compost soil at different measurements 10 μm to 50 μm; Figure S3. (a,b). Block diagram of the setup used for potential-static electrical measurements of ammonium fuel cell in a coin cell geometry and for Multifunctional ammonium fuel cell. (a) Keithley (IV) Electrical Measurement Setup for CS-AFC coin Cell small size design adjusted according to the voltage window, (b) Electrical Measurement Setup for multifunctional bulk compost soil Ammonium Fuel Cell (CS-AFC) design for big commercial model; Figure S4. Effect of bipolar CV studies between Cu/Cu and Gr/Gr electrodes and its introductory study checked without membrane (a) CS-AFC (b) EIS studies shows that in comparison between G/G and Cu/Cu electrodes and the overall performance of Cu/Cu combination is best for power generation; Figure S5. The comparison survey data of different soil fuel cells in survey with power density; Figure S6. Showing the difference between the auto calved studies where the growth of the microbes is absent. In the 8(**a**) from 0 to 120 h and 8(**b**) showing the presence of the microbial growth from 0 to 120 h.
