# Supplementary material for: High Power Generation with Reducing Agents Using Compost Soil as a Novel Electrocatalyst for Ammonium Fuel Cells
Source: Nanomaterials (Basel). 2022 Apr 9;12(8):1281. doi: 10.3390/nano12081281 (PMC9029104; doi:10.3390/nano12081281)
Supplement: Supplementary file 1 [file nanomaterials-12-01281-s001.zip › nanomaterials-1600576-supplementary.pdf]

Supplementary Materials

High Power Generation with Reducing Agents Using Compost Soil as a Novel Electrocatalyst for Ammonium Fuel Cells

Verjesh Kumar Magotra [1], Seung Joo Lee [1], Tae Won Kang [1], Akbar I. Inamdar [2], Deuk Young Kim [2], Hyunsik Im [2] and Hee Chang Jeon [1,*]

[1] Nano Information Technology Academy, Dongguk University, Jung-Gu, Seoul 100715, Korea; birju.srm@gmail.com (V.K.M.); leesj@dongguk.edu (S.J.L.); twkang@dongguk.edu (T.W.K.)
[2] Division of Physics and Semiconductor Science, Dongguk University, Jung-Gu, Seoul 100715, Korea; akbarphysics2002@gmail.com (A.I.I.); dykim@dongguk.edu (D.Y.K.); hyunsik7@dongguk.edu (H.I.)
* Correspondence: hcjeon@dongguk.edu

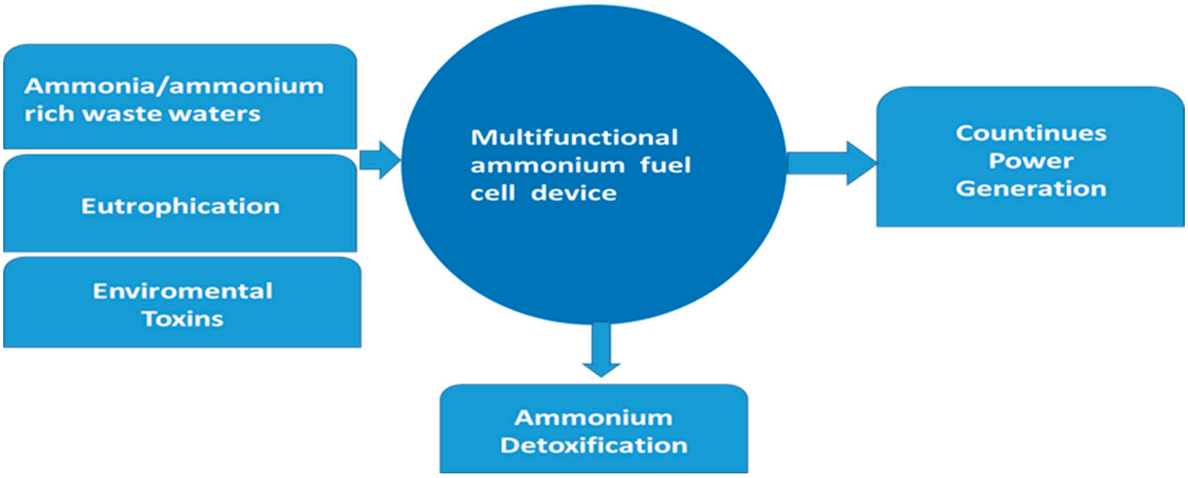

Figure S1. The schematic diagram showing the vision of this paper is shown in Figure S1. This figure highlights the use of the multifunctional device for environmental detoxification and continuous power generation.

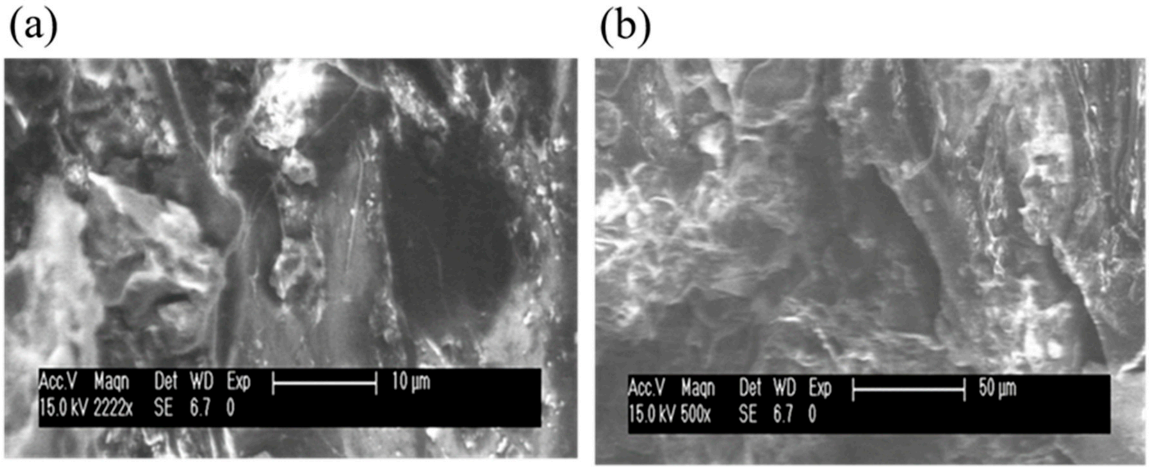

Figure S2. (**a**,**b**) Explain the SEM image to study the surface texture of the compost soil at different measurements 10 μm to 50 μm.

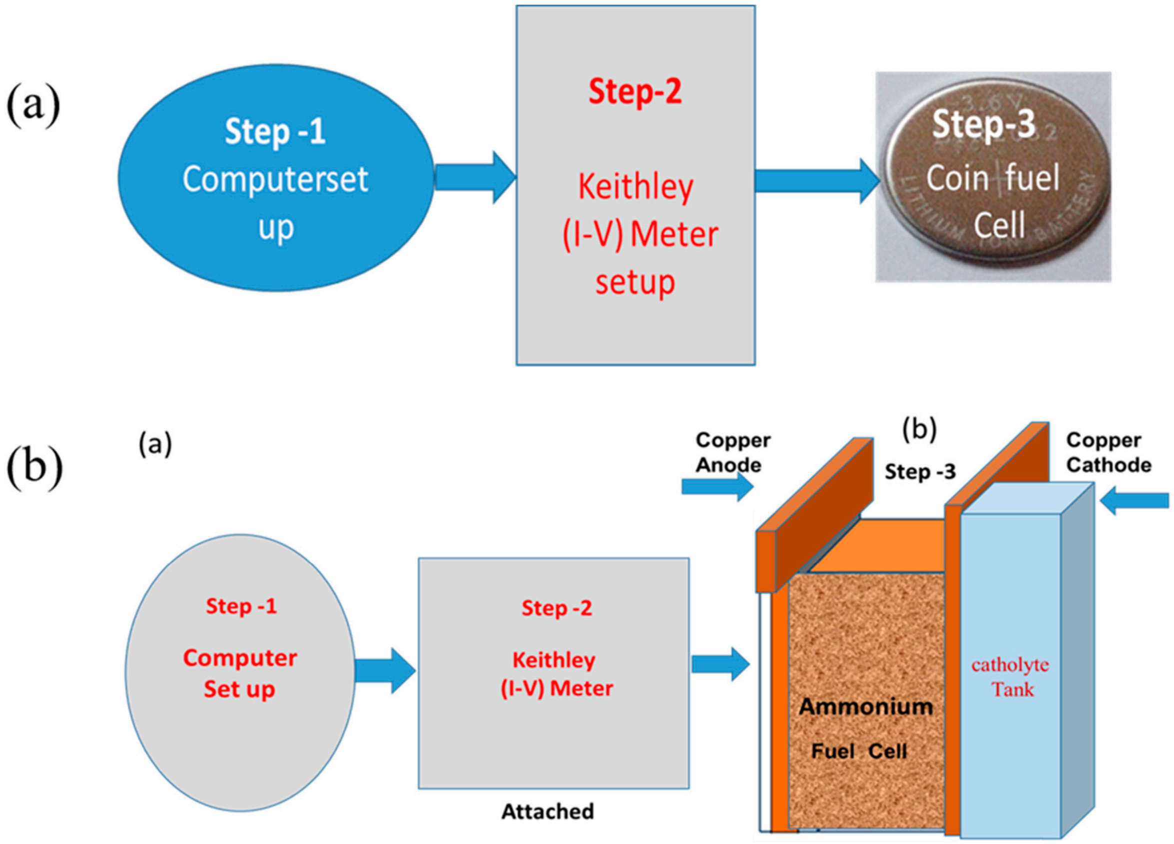

Figure S3. (**a**,**b**). Block diagram of the setup used for potential-static electrical measurements of ammonium fuel cell in a coin cell geometry and for Multifunctional ammonium fuel cell. (**a**) Keithley (IV) Electrical Measurement Setup for CS-AFC coin Cell small size design adjusted according to the voltage window, (**b**) Electrical Measurement Setup for multifunctional bulk compost soil Ammonium Fuel Cell (CS-AFC) design for big commercial model.

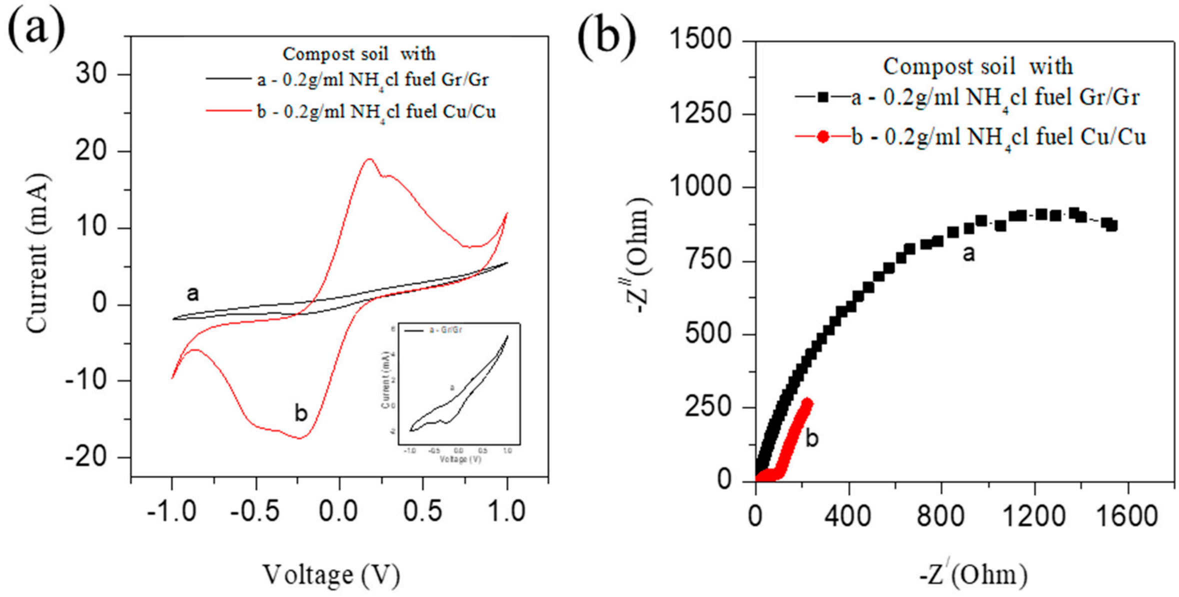

Figure S4. Effect of bipolar CV studies between Cu/Cu and Gr/Gr electrodes and its introductory study checked without membrane (**a**) CS-AFC (**b**) EIS studies shows that in comparison between G/G and Cu/Cu electrodes and the overall performance of Cu/Cu combination is best for power generation.

S/No	Type of Electrode	Fuel used	Power density	MFC Type	Referecne s	Year
1	Gr/Gr	Urea	3mw/m2	Soil	26	2020
2	Gr/Gr	Urea	18mw/m^2	Soil	34	2020
3	Gr/Gr	Ammonium Chloride	0.78 mA	Liquid state	2	2007
4	Gr/Gr	Glucose	32mW/m²	soil	15	2010
5	Cu/Cu	Ammonium chloride	108 mW/m²	Soil	4	2018
6	Gr.Felt/Gr	Waste water	45.04 mW/m²	Soil	52	2019
**Data with Reducing Potassium Ferrycyanide**						
7	Gr/carbon cloth	Fuel	2.3 mW/m²	Liquid state	51	2006
8	Gr/Carbon brush	Wastwater	260 mW/m²	Liquid state	34	2020
9	Carbon/Gr paper	Wastewater	181.78 mW/m²	Liquid state	35	2012
10	Gr/Gr	Acetate	166 mW/m²	Liquid state	24	2017
11	Cu/Cu	Ammonium chloride	1785.20 mW/m²	Compost soil	Our work	Present work

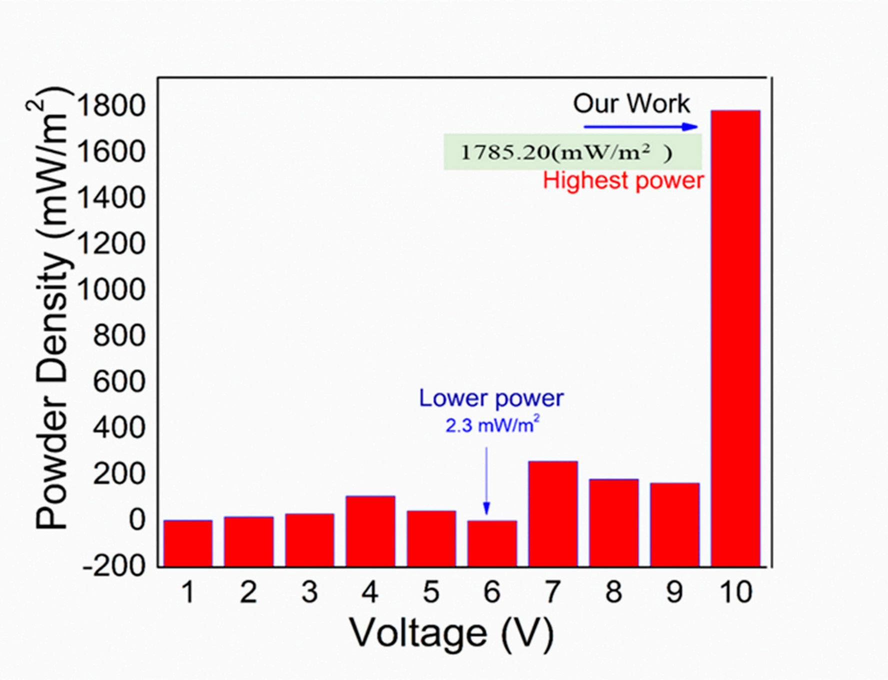

Figure S5. The comparison survey data of different soil fuel cells in survey with power density.

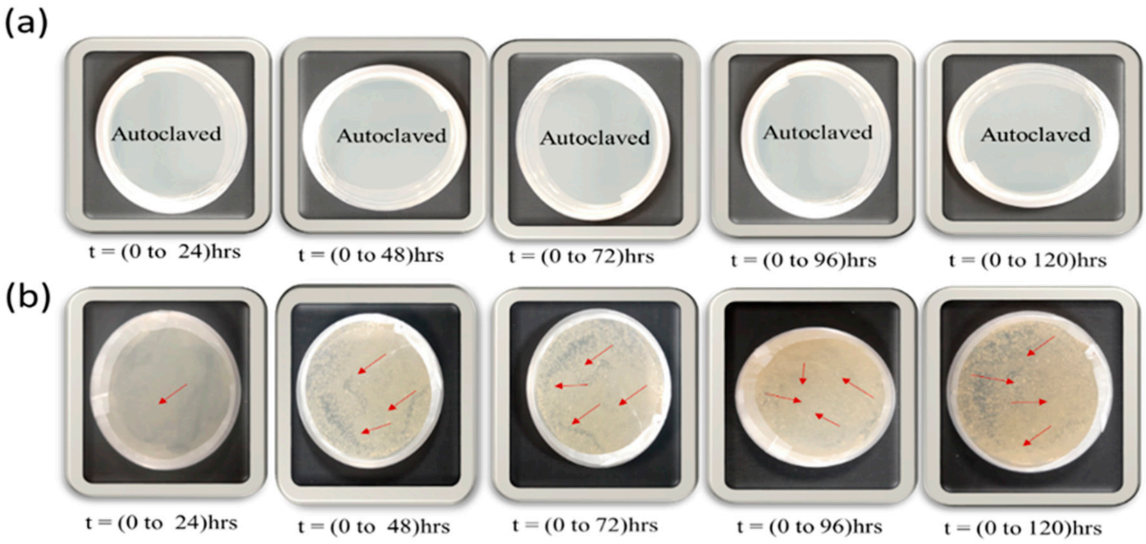

Figure S6. Showing the difference between the auto calved studies where the growth of the microbes is absent. In the 8(**a**) from 0 to 120 h and 8(**b**) showing the presence of the microbial growth from 0 to 120 h.
